# Apigenin Protects Mouse Retina against Oxidative Damage by Regulating the Nrf2 Pathway and Autophagy

**DOI:** 10.1155/2020/9420704

**Published:** 2020-05-13

**Authors:** Yuanzhong Zhang, Yan Yang, Haitao Yu, Min Li, Li Hang, Xinrong Xu

**Affiliations:** ^1^Department of Ophthalmology, Jiangsu Province Hospital of Chinese Medicine (Affiliated Hospital of Nanjing University of Chinese Medicine), Nanjing 210029, China; ^2^Department of Ophthalmology, The Second Affiliated Hospital of Nanjing University of Chinese Medicine, Nanjing 210017, China; ^3^School of Pharmacy, Nanjing University of Chinese Medicine, Nanjing 210023, China; ^4^Department of Ophthalmology, Liyang Branch of Jiangsu Province Hospital of Chinese Medicine, Liyang 213300, China

## Abstract

Oxidative stress is a critical factor in the pathology of age-related macular degeneration (AMD). Apigenin (AP) is a flavonoid with an outstanding antioxidant activity. We had previously observed that AP protected APRE-19 cells against oxidative injury *in vitro*. However, AP has poor water and fat solubility, which determines its low oral bioavailability. In this study, we prepared the solid dispersion of apigenin (AP-SD). The solubility and dissolution of AP-SD was significantly better than that of the original drug, so the oral bioavailability in rats was better than that of the original drug. Then, the effects of AP-SD on the retina of a model mouse with dry AMD were assessed by fundus autofluorescence (FAF), optical coherence tomography (OCT), and electron microscopy; the results revealed that AP-SD alleviated retinopathy. Further research found that AP-SD promoted the nuclear translocation of Nrf2 and increased expression levels of the Nrf2 and target genes HO-1 and NQO-1. AP-SD enhanced the activities of SOD and GSH-Px and decreased the levels of ROS and MDA. Furthermore, AP-SD upregulated the expressions of p62 and LC3II in an Nrf2-dependent manner. However, these effects of AP-SD were observed only in the retina of Nrf2 WT mice, not in Nrf2 KO mice. In addition, the therapeutic effect of AP-SD was dose dependent, and AP did not work. In conclusion, AP-SD significantly enhanced the bioavailability of the original drug and reduced retinal oxidative injury in the model mouse of dry AMD *in vivo*. The results of the underlying mechanism showed that AP-SD upregulated the expression of antioxidant enzymes through the Nrf2 pathway and upregulated autophagy, thus inhibiting retinal oxidative damage. AP-SD may be a potential compound for the treatment of dry AMD.

## 1. Introduction

Age-related macular degeneration (AMD) is a leading cause of vision loss in the elderly. Clinically, AMD falls into two categories: dry AMD and wet AMD. The dry AMD presents as retinal pigment proliferation or depigmentation, drusen, and advanced geographic atrophy. The wet AMD is characterized by choroidal neovascularization (CNV), leading to retinal exudation and hemorrhage, and eventually severe visual impairment. In recent years, the treatment of wet AMD has made significant progress due to the widespread use of antivascular endothelial growth factor (VEGF) drugs [1]. Currently, there are no effective treatment options for dry AMD.

A large number of evidences indicate that oxidative damage of retinal pigment epithelium (RPE) is a main etiology of AMD. The RPE cell has abundant mitochondria and produces a large amount of reactive oxygen species (ROS) in a high-oxygen environment [2]. In addition, one of the major functions of RPE is the phagocytosis and degradation of photoreceptor outer segments (POS). POS is rich in unsaturated fatty acids, and the part being not degraded by RPE forms lipofuscin, which increases ROS generation while exposed to light [3]. The nuclear factor E2-related factor 2 (Nrf2) pathway is a primary system employed by the RPE to neutralize oxidative stress and maintain cellular homeostasis [4]. In a physiological condition, Nrf2 is constitutively targeted for degradation by the multisubunit, E3 ubiquitin lipase KEAP1. Oxidative stress dissociates KEAP1 and stabilizes Nrf2. The transcription factor rapidly translocates to the nucleus, heterodimerizes with Maf proteins, and binds to the antioxidant response elements (AREs) in the promoters of its cognate target genes, inducing the expression of genes encoding heme oxygenase-1 (HO-1), quinone oxidoreductase-1 (NQO-1), glutathione peroxidase (GSH-Px), superoxide dismutase (SOD), and catalase (CAT). These enzymes can quickly scavenge ROS and protect the body from injuries caused by active substances or toxic substances [5]. Thus, the activation of the Nrf2 pathway could reduce the oxidative damage of RPE, suggesting that the Nrf2 pathway is a potential target for AMD treatment [6].

As Nrf2 pathway does, autophagy keeps cellular homeostasis in situations of oxidative stress; therefore, its failure is associated with aging and many diseases, such as cancer, cardiovascular diseases, and neurodegenerative diseases [7]. The impairment of autophagy has been shown to be one cause of the development and progression of AMD [8]. Impaired autophagy significantly reduced the degradation of POS, leading to a deposit of undegraded POS under RPE, and finally an increase of ROS generation [9, 10]. The autophagy is increased and the Nrf2 pathway is activated at the same time while the cell is in oxidative stress. Polyunsaturated fatty acids rapidly increase the generation of ROS in RPE and upregulate the expression of Nrf2 and SQSTM1/p62 proteins [11]. Studies have shown that many inducers of the Nrf2 pathway can also induce autophagy [12–14]. Correspondingly, a deficiency of Nrf2 reduces SQSTM1/p62 expression [15]. Clearly, there is a strong correlation between autophagy and Nrf2 pathways, which makes sense for AMD.

Apigenin (AP) is a bioactive flavonoid which is obtained from several fruits and vegetables. AP has been reported to exhibit antioxidant, anti-inflammatory, and anticancer activities [16, 17]. Our previous study has shown that AP exhibited protective effects on ARPE-19 cells against tert-butyl hydroperoxide- (t-BHP-) induced oxidative injury, which were associated with its antioxidant properties dependent upon the activation of Nrf2 signaling [18]. However, AP has the disadvantages of poor water and fat solubility and low bioavailability. Studies have indicated that the preparation of solid dispersion could significantly increase the solubility and dissolution rate of drugs, which can further enhance the bioavailability of drugs [19, 20]. In the current study, we prepared the solid dispersion of apigenin (AP-SD), determined its bioavailability in rats, and evaluated its protective effects on retinal injury induced by oxidative stress in Nrf2 WT and Nrf2 KO mice.

## 2. Materials and Methods

### 2.1. Chemicals, Reagents, and Antibodies

Apigenin was from Sigma Chemical (St. Louis, MO, USA). Polyvinylpyrrolidone (PVP K30) was from J&K Chemical Ltd. (Shanghai, China). Hydroquinone (HQ) was from Alfa Aesar (Heysham, Lancashire, UK). The primary antibodies used in western blot were obtained from the following source: Nrf2, HO-1, NQO-1, p62, GAPDH, and Lamin B (Abcam, UK) and LC3 (Cell Signaling Technology, USA); the secondary antibody Goat Anti-Rabbit IgG/HRP was from (Abcam, UK).

### 2.2. Preparation of AP-SD

With the orthogonal experiment, we assigned PVP K30 as the carrier to prepare the solid dispersion of AP, the mass ratio of AP and PVK V30 is 1: 9, and anhydrous ethanol is used as the reaction solvent to prepare AP-SD. Briefly, AP and PVK V30 at a mass ratio of 1 : 9 were dissolved in absolute ethanol of adequate volume with full mixing. After the mixture was evaporated in vacuo and vacuum drying at 50°C, the AP-SD was obtained for experiments.

We then determined the standard curve of AP-SD. The chromatographic conditions were Waters 600 C_l8_ column, methanol-0.1% CH_3_COONa solution (*v*/*v* 60 : 40) as the mobile phase, 337 nm wavelength, 1.0 mL/min flow velocity, 40°C column temperature, and 10 *μ*L sample size. The control solution was prepared according to following the procedures. AP of 10 mg was dissolved in ethanol and volumed in 50 mL volumetric flask, and thereby the AP control solution at a concentration of 1 mg/mL was obtained. Anhydrous ethanol was used to dilute the control solution to yield a series of solutions at 1, 2, 4, 8, 16, and 32 *μ*g/mL. Each solution of 10 *μ*L was injected into a high-performance liquid chromatograph (HPLC, Waters 600, USA) for measurement of the peak area. Concentrations of AP ([*C*]) were marked at the horizontal ordinate and peak areas (*A*) at the longitudinal coordinate giving the standard curve equation: *A* = 32786.6 × [*C*] − 160.4 (*R* = 0.9991), linear range 0.56-32.7 *μ*g/mL.

### 2.3. Determination of Solubility

AP and AP-SD (equivalent to 10 mg of AP) were precisely weighted and added into 100 mL conical flask, and then, distilled water/chloroform of 20 mL was added. The solutions were incubated in a 25°C thermostatic oscillator for 24 hr. Each sample of 5 mL was filtered through a 0.45 *μ*m microporous membrane. The successive filtrate of 10 *μ*L was subjected to HPLC analyses. Control solution of 10 *μ*L at 8 *μ*g/mL was spontaneously analyzed by HPLC. The equilibrium solubility of both samples in water and chloroform was measured, respectively, based on the peak area.

### 2.4. Determination of Dissolution Rate

AP and AP-SD (both containing 10 mg pure AP) were evenly dispersed in the dissolution medium (phosphate buffer saline, pH 7.4, 37°C, *V* = 900 mL) and centrifuged at 100 r/min. Each sample of 5 mL was collected at the time points of 5, 10, 15, 20, 30, 45, 60, and 90 min, respectively, and subjected to filtration. The successive filtrate of 1 mL was volumed in 10 mL volumetric flask with distilled water. HPLC analyses were used, and mass concentrations were calculated through introducing a peak area into the standard curve equation followed by calculation of the cumulative dissolution percentage.

### 2.5. Determination of Plasma Concentrations of AP

Twelve Sprague Dawley rats (Model Animal Research Centre of Nanjing University (Nanjing, China) were randomly divided into two groups: the AP and AP-SD groups (*n* = 6). Rats of both groups were intragastrically administrated with corresponding drugs containing pure AP at 50 mg/kg. Blood samples were collected from orbit at time points of 0.25, 0.5, 1, 2, 4, 8, 12, and 24 hr of each rat. After adding heparin, blood samples were centrifuged at 3000 r/min for 15 min, and the supernatants were collected for examination. 200 *μ*L plasma was mixed with 600 *μ*L of silybin solution (internal standard, 13.3 *μ*g/mL in methanol) and vortex mixed for 5 min. After centrifugation for 5 min at 10000 r/min, the supernatant was collected. The rest of the residue was extracted with another 300 *μ*L of methanol by vortex mixing for 5 min and centrifuged for 5 min at 10000 r/min again, and the obtained supernatant was pooled together with the previous supernatant. Then, the total supernatant was dried under a nitrogen flow at 35°C and the obtained residues were reconstituted with 200 *μ*L of methanol. After centrifugation for another 5 min at 10000 r/min, an aliquot of 20 *μ*L supernatant was analyzed by HPLC for determining drug concentrations. Maximum plasma concentration (*C*_max_) and time to reach maximum concentration (*T*_max_) were obtained directly from the concentration-time curve; area under the plasma concentration-time curve from zero to the time of the final sample measurement (AUC_0-24_) was calculated using the statistical software GraphPad Prism 5 (GraphPad Software Inc., CA, USA).

### 2.6. Animal Experimental Procedures

C57BL/6 mice (6 months old, bodyweight 25-33 g, Nrf2 WT and KO) were obtained from the Model Animal Research Centre of Nanjing University (Nanjing, China). All mice were domesticated under a 12 h light/dark cycle at a controlled temperature (25°C) with free access to food and tap water. Nrf2 WT mice were divided into 6 groups: the aging control, model control, AP 60 (60 mg/kg), and AP-SD groups (20, 40, and 60 mg/kg). Nrf2 KO mice were divided into 3 groups: the aging control, model control, and AP-SD 60 groups (60 mg/kg). The doses were defined by the content of pure AP in the solid dispersion and were determined by preliminary experiments. Animals were treated according to the following procedures: aging control mice were fed normal diet for 9 months; model control mice were fed normal diet during months 1-3, high-fat diet and intake of HQ dissolved in the drinking water (0.8%) during months 4-6, normal diet and intragastric administration with 0.5% CMC-Na suspension daily during months 7-9. For the treated group mice, model control mice were intragastrically administrated with corresponding drugs (suspended in 0.5% CMC-Na) at the last 3 months. All experimental procedures were approved by the institutional and local committee for the care and use of animals, and all animals received humane care according to the National Institutes of Health (USA) guidelines.

### 2.7. Fundus Autofluorescence

The mice were anesthetized using a ketamine (120 mg/kg body weight) and xylazine (8 mg/kg body weight) intraperitoneal injection, and the pupil was fully dilated with tropicamide phenylephrine eye drops (Kanda Pharmaceutical, Japan). The mouse was placed on the table with its head in the chinrest. To maintain corneal hydration and improve image quality, the viscoelastic material (Viscoat, Alcon-Couvreur, Belgium) was smeared on the cornea covering with a coverslip. A 90D noncontact slit lamp lens was fixated directly in front of the confocal scanning laser ophthalmoscope (Heidelberg Engineering, Heidelberg, Germany). Fluorescence was excited using a 488 nm argon laser or a 790 nm diode laser. As Charbel Issa did [21], images were recorded using the ART mode for the quantitative analysis of fundus autofluorescence (FAF); the mean grey level on mouse FAF images was measured using ImageJ software (version 1.52, NIH, Bethesda, USA).

### 2.8. Optical Coherence Tomography

Like the FAF assessment, the animal was anesthetized, the pupil fully dilated, and the viscoelastic material was smeared on the cornea covering with a coverslip; then, a 90D noncontact slit lamp lens was placed in front of an optical coherence tomography (OCT) device (Cirrus HD-OCT 4000, Carl Zeiss Meditec, Dublin, CA); cross-sectional images of the retina were undertaken. The average thickness of the mouse retina was obtained using the macular cube scan mode according to the methods reported by Song [22].

### 2.9. Transmission Electron Microscopy

Eyeballs were fixed in 4% paraformaldehyde for 20 min. The cornea, lens, and vitreous were removed. The yye wall tissue (2 × 4 mm) was excised from the bilateral area of the optic disc and fixed with glutaral/osmic acid, coated with epoxy resins, and sectioned. After double staining with uranyl acetate and lead citrate, the sections were examined with transmission electron microscope (Tecnai G2 Spirit BioTWIN; FEI, Hillsboro, OR, USA) and images were taken. The area of the sediment beneath RPE was determined according to the methods reported by Edward [23]. Briefly, images were opened in ImageJ software and calibrated using the embedded scale bar. The region of the sediment was drawn using free selection tool, and the area was measured. The thickness of the Bruch membrane (BrM) was directly measured by electron microscopy.

### 2.10. Measurements of ROS, MDA, and Antioxidant Enzymes

The retina was isolated, and tissue homogenates were prepared via centrifugation at 4°C, 3000 r/min for 10 min. Levels of ROS were determined using the DCFH-DA method. Levels of MDA were measured using the thiobarbituric acid method. In addition, activities of SOD and GSH-PX were measured using the corresponding enzyme-linked immunosorbent assay kits at the wavelength of 450 and 412 nm, respectively. Kits above were from Nanjing Jiancheng Bioengineering Institute (Nanjing, China), and experiments were performed according to the instructions provided by the manufactures.

### 2.11. Western Blot Analysis

The mouse retina/choroid tissue was lysed in the RIPA buffer containing a protease inhibitor.

For examining the Nrf2 expression, nuclear and cytoplasmic proteins were separated using a Bioepitope Nuclear and Cytoplasmic Extraction Kit (Bioworld Technology, St. Louis Park, MN, USA) following the manufacturer's guidelines. Proteins (50 *μ*g/well) were separated by SDS-polyacrylamide gel and transferred to a PVDF membrane (Millipore, Burlington, MA, USA). After blocking with 5% skim milk in Tris-buffered saline containing 0.1% Tween 20, the membranes were incubated with primary corresponding primary antibodies. The blots were then incubated with a secondary antibody (anti-rabbit horseradish peroxidase-conjugate/anti-mouse HRP-conjugate), and the protein bands were visualized using a chemiluminescence reagent (Millipore, Burlington, MA, USA). Equivalent loading was confirmed using an antibody against GAPDH for total proteins and against Lamin B for nuclear proteins. The levels of target protein bands were densitometrically determined using Quantity One 4.4.1.

### 2.12. Statistical Analysis

Data were presented as mean ± SD, and results were analyzed using GraphPad Prism 5 software. The significance of difference was determined by Student's *t*-test for comparison between two groups and one-way ANOVA with post hoc Tukey's test for comparison between multiple groups. A value of *p* < 0.05 was considered to be statistically significant.

## 3. Results

### 3.1. AP-SD Increases the Solubility and Dissolution of AP In Vitro

**Figure 1:**
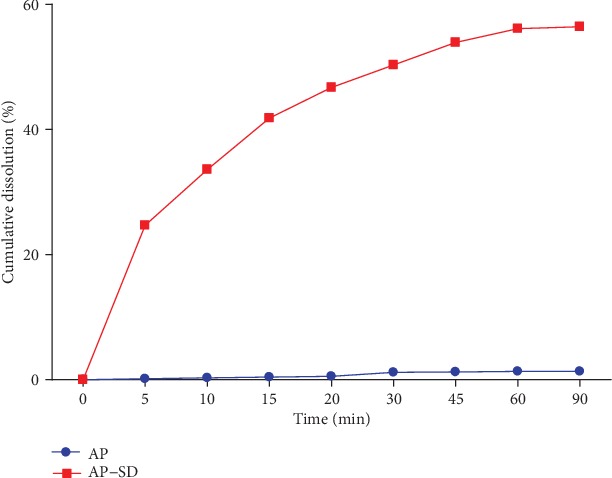
AP-SD had significantly higher cumulative dissolution rates than AP. Determination of cumulative dissolution rates of AP and AP-SD at indicated time points. Data are means ± standard deviation (*n* = 6).

We determined the equilibrium solubility of AP and AP-SD in water and chloroform. The results showed that the equilibrium solubility of AP-SD in both water and chloroform was significantly higher than that of AP (Table 1). Determination of the dissolution rate showed that AP-SD had significantly higher cumulative dissolution rates than AP at each time points (Figure 1).

### 3.2. AP-SD Enhances the Absorption of AP In Vivo

**Figure 2:**
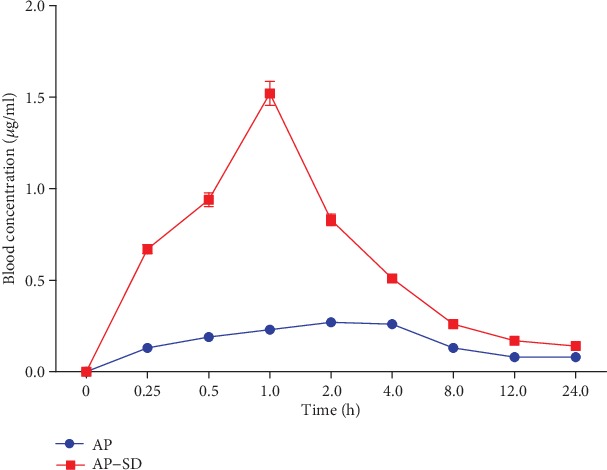
AP-SD had better oral bioavailability than AP. Determination of blood concentrations of AP and AP-SD at indicated time points in rats. Data are means ± standard deviation (*n* = 6).

We obtained the plasma concentration-time curve of AP and AP-SD in rats (Figure 2). *C*_max_, *T*_max_, and AUC_0~24_ for AP and AP-SD were calculated with ImageJ software (Table 2). The results showed that AP-SD had better bioavailability than AP.

### 3.3. AP-SD Alleviated Pathological Changes of the Retina

**Figure 3:**
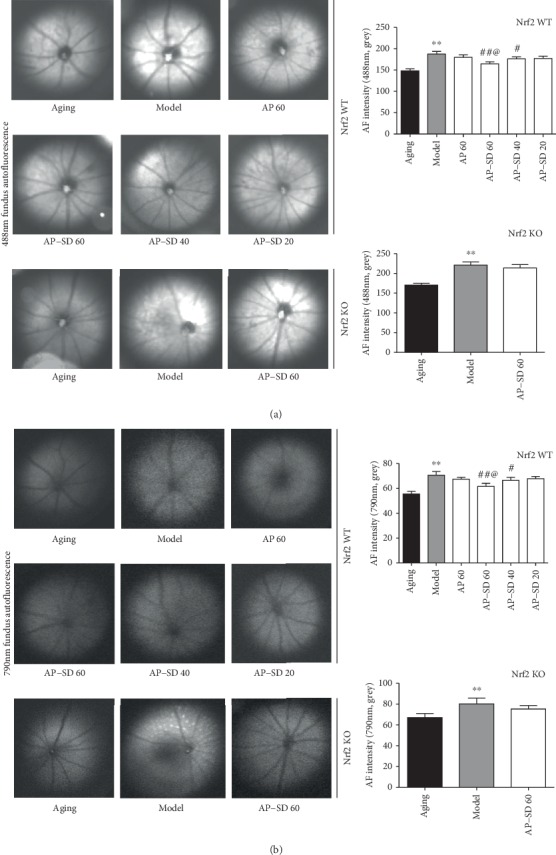
Representative images of fundus AF excited at 488 (a) and 790 nm (b) in Nrf2 WT and KO mice. AF intensity in model mice was significantly enhanced compared with that in aging mice, and in Nrf2 KO mice higher than in Nrf2 WT mice. AF intensity was decreased after treatment with AP-SD in Nrf2 WT mice, but not in Nrf2 KO mice. ^※※^*p* < 0.01, model control versus aging control; ^#^*p* < 0.05, ^##^*p* < 0.01, AP-SD versus model control; ^@^*p* < 0.05, AP-SD 60 versus AP-SD 40. Data are means ± standard deviation (*n* = 5).

**Figure 4:**
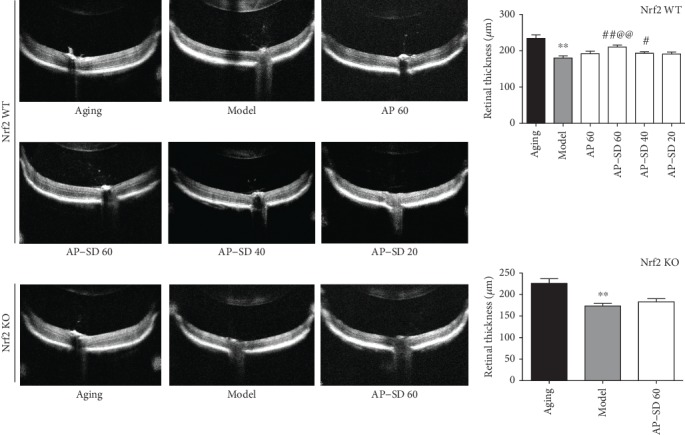
Representative images of OCT in Nrf2 WT and KO mice. Outer layer structure of the retina was unclear and the retina was thinner in model mice compared with aging mice, more significant in Nrf2 KO mice. Treatment with AP-SD dose dependently restored the retinal structure in Nrf2 WT mice and did not in Nrf2 KO mice. ^※※^*p* < 0.01, model control versus aging control; ^#^*p* < 0.05, ^##^*p* < 0.01, AP-SD versus model control; ^@@^*p* < 0.01, AP-SD 60 versus AP-SD 40. Data are means ± standard deviation (*n* = 5).

In clinical, fundus AF and OCT are the most used noninvasive means for monitoring of dry AMD [24, 25]. Fundus AF generated with wavelength between 500 and 750 nm is dominated by RPE lipofuscin, a complex mixture of fluorophores being accumulated in the RPE after phagocytosis of POS [26]. Therefore, AF intensity indicates the level of lipofuscin in vivo in the RPE. Spectral-domain- (SD-) OCT provides high-quality, cross-sectional images of the retina including RPE with resolution approaching histology performed with light microscopy [27]. To evaluate the therapeutic effects of AP-SD, we established a dry AMD mouse model that mimics three risk factors for AMD in humans: aging, hyperlipidemia, and smoking (HQ is abundant in cigarette smoke) [28]. HQ, an electrophilic, could inhibit the binding of BACH1 with Nrf2, thus activating the Nrf2 pathway [29]. Our results showed that AF intensity in model mice was significantly enhanced compared with that in aging mice, and in Nrf2KO mouse higher than in Nrf2WT mice. AF intensity was attenuated after treatment with AP-SD in Nrf2WT mice (Figures 3(a) and 3(b)). Correspondingly, the images of OCT scanning showed that the outer layer structure of the retina including photoreceptors, RPE, and Bruch membrane (BrM) became unclear, and the retina was thinner in model mice compared with aging mice, more significant in Nrf2 KO mice than in Nrf2 WT mice (Figure 4). Treatment with AP-SD could restore the retinal structure in Nrf2WT mice.

**Figure 5:**
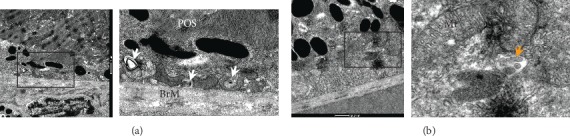
Transmission electron microscopy showed that there were undigested phagosomes (a, white arrow) and autophagosome (b, orange arrow) present in the RPE of the model mouse. POS: photoreceptor outer segment; Mt: mitochondria; BrM: Bruch membrane.

**Figure 6:**
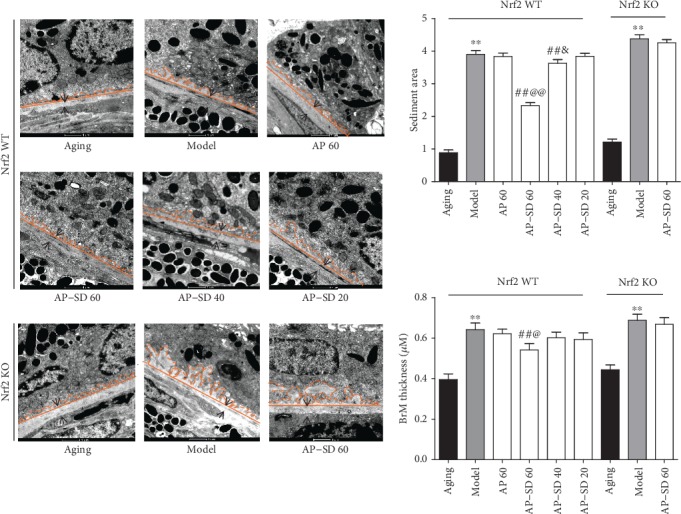
Representative images of TEM in Nrf2 WT and KO mice. There were obvious sediments under RPE (orange area) and thickened BrM (black arrow) in model mice compared to the aging mice, and it is more severe in Nrf2 KO mice. AP-SD reduced the area of the sediment and thinned BrM in Nrf2 WT mice and not in Nrf2 KO mice. ^※※^*p* < 0.01, model control versus aging control; ^##^*p* < 0.01, AP-SD versus model control; ^@^*p* < 0.05, ^@@^*p* < 0.01, AP-SD 60 versus AP-SD 40; ^&^*p* < 0.05, AP-SD 40 versus AP-SD 20. Data are means ± standard deviation (*n* = 5).

We further observed the retinal ultrastructure; the results demonstrated that there were undigested phagosomes (Figure 5(a)) and autophagosome (Figure 5(b)) present in the RPE of the model mouse. There were obvious sediments under RPE and thickened BrM in the model mice compared to the aging mice, and it is more severe in Nrf2 KO mice. AP-SD reduced the area of the sediment and thinned BrM in Nrf2WT mice (Figure 6). In particular, AP-SD only alleviated retinopathy in Nrf2 WT mice and had no effects in Nrf2 KO mice. In addition, the therapeutic effect of AP-SD was dose dependent, and AP did not work. Collectively, these data indicated that AP-SD more potently improved pathological changes of the retina in Nrf2 WT model mice with dry AMD.

### 3.4. AP-SD Induced Nrf2 Nuclear Translocation and Increased Antioxidant Gene Expression

**Figure 7:**
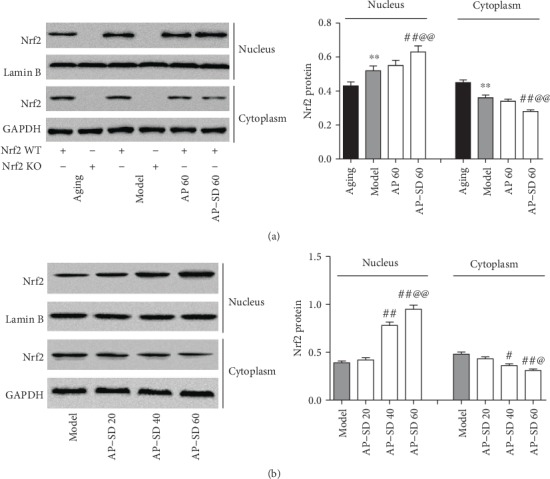
AP-SD promoted Nrf2 nuclear translocation. (a) The level of nuclear Nrf2 was increased and the level of cytoplasmic Nrf2 was reduced in model mice compared with those in aging mice. (b) AP-SD decreased the level of cytoplasmic Nrf2 and increased the level of nuclear Nrf2. ^※※^*p* < 0.01, model control versus aging control; ^##^*p* < 0.01, AP-SD versus model control; ^@^*p* < 0.05, ^@@^p < 0.01, AP-SD 60 versus AP-SD 40. Data are means ± standard deviation (*n* = 5).

**Figure 8:**
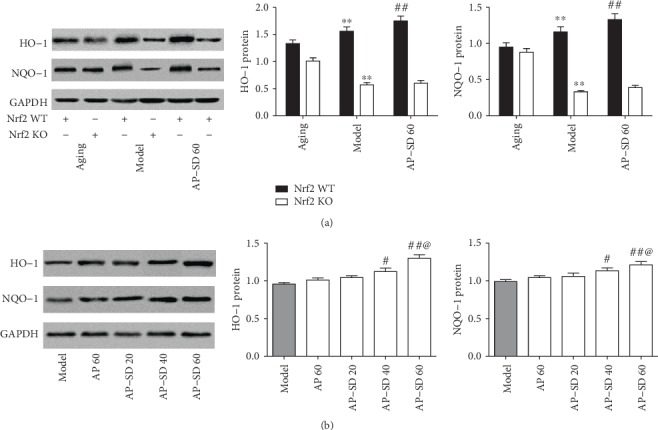
AP-SD upregulated the expressions of the targeted genes of Nrf2. (a) The expressions of HO-1 and NQO-1 were upregulated in the Nrf2 WT model mice and downregulated in Nrf2 KO model mice, compared with those in aging mice. (b) AP-SD increased the expressions of HO-1 and NQO-1 in Nrf2 WT mice but not in Nrf2 KO mice. ^※※^*p* < 0.01, model control versus aging control;^#^*p* < 0.05, ^##^*p* < 0.01, AP-SD versus model control; ^@^*p* < 0.05, AP-SD 60 versus AP-SD 40. Data are means ± standard deviation (*n* = 5).

To explore the underlying mechanism for AP-SD reducing retinopathy in model mice with dry AMD, we first assessed the effects of AP-SD on Nrf2 pathway. As shown in Figure 7(a), the level of nuclear Nrf2 was increased and the level of cytoplasmic Nrf2 was reduced in model mice compared with those in aging mice. AP-SD decreased the level of cytoplasmic Nrf2 and increased the level of nuclear Nrf2 in a dose-effect manner, indicating that AP-SD promoted Nrf2 nuclear translocation (Figure 7(b)). Furthermore, we examined the ability of AP-SD to upregulate the expressions of the targeted genes of Nrf2, upon its increased nuclear translocation. The expressions of HO-1 and NQO-1 were upregulated in the Nrf2 WT model mice and downregulated in the Nrf2 KO model mice, compared with those in aging mice (Figure 8(a)). AP-SD dose dependently increased the expressions of HO-1 and NQO-1 in Nrf2 WT mice but had no effects in Nrf2 KO mice as expected (Figure 8(b)).

### 3.5. AP-SD Restored Activities of SOD and GSH-Px Enzyme and Decreased Levels of SOD and MDA

**Figure 9:**
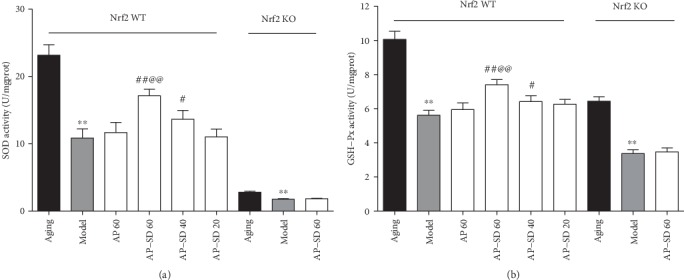
AP-SD restored the activities of SOD and GSH-Px. The activities of SOD (a) and GSH-Px (b) were decreased in Nrf2 WT model mice and more remarkable in Nrf2 KO model mice. AP-SD restored the activities of SOD and GSH-Px in Nrf2 WT mice, not in Nrf2 KO mice. ^※※^*p* < 0.01, model control versus aging control; ^#^*p* < 0.05, ^##^*p* < 0.01, AP-SD versus model control; ^@@^*p* < 0.01, AP-SD 60 versus AP-SD 40. Data are means ± standard deviation (*n* = 5).

**Figure 10:**
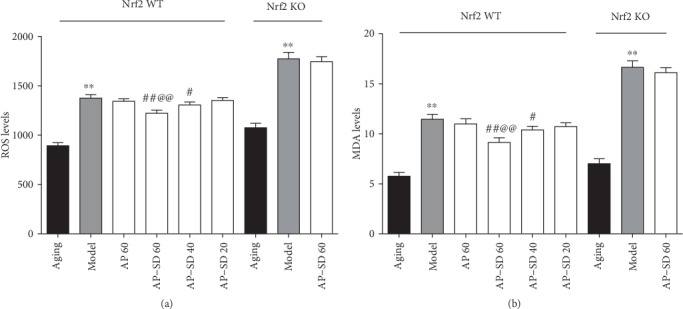
AP-SD decreased the ROS and MDA levels. The levels of ROS (a) and MDA (b) were significantly elevated in Nrf2 WT and Nrf2 KO model mice, but higher in Nrf2 KO mice. AP-SD decreased the ROS and MDA levels in Nrf2 WT mice, not in Nrf2 KO mice. ^※※^*p* < 0.01, model control versus aging control; ^#^*p* < 0.05, ^##^*p* < 0.01, AP-SD versus model control; ^@@^*p* < 0.01, AP-SD 60 versus AP-SD 40. Data are means ± standard deviation (*n* = 5).

We measured the activity of SOD and GSH-Px enzyme in mouse, two important antioxidant enzymes of the retina. The results showed that the activities of SOD and GSH-Px were significantly decreased in Nrf2 WT model mice and are more remarkable in Nrf2 KO model mice. AP-SD dose dependently restored the activities of SOD and GSH-Px in Nrf2 WT mice, not in Nrf2 KO mice (Figures 9(a) and, 9(b)). ROS is a by-product of cellular oxidative phosphorylation and its level is consistent with oxidative stress. ROS causes lipid peroxidation of the biomembrane, which leads to the production of a large amount of MDA [2]. Therefore, we examined the levels of ROS and MDA in the mouse retina. The results showed that the levels of ROS and MDA were significantly elevated in Nrf2 WT and Nrf2 KO model mice, but higher in Nrf2 KO mice. AP-SD decreased the ROS and MDA levels in dose-effect manner in Nrf2 WT mice, not in Nrf2 KO mice (Figures 10(a) and 10(b)).

### 3.6. AP-SD Increased p62 Protein Expression and Regulated Autophagy in an Nrf2-Dependent Manner

**Figure 11:**
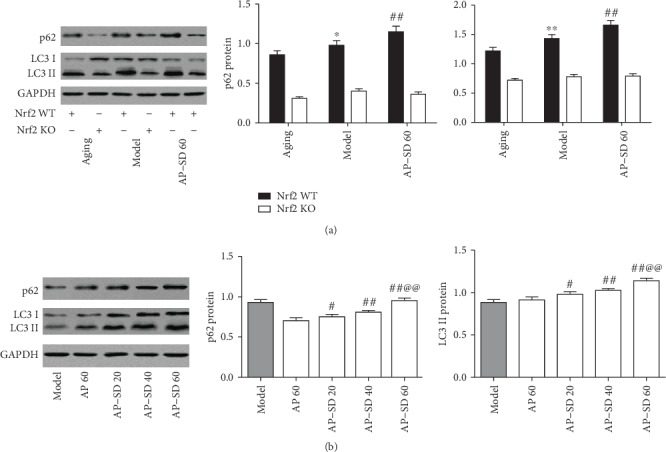
AP-SD regulated autophagy. (a) The expressions of p62 and LC3 II were upregulated in Nrf2 WT model mice, but not in Nrf2 KO model mice, compared with those in aging mice. (b) AP-SD increased the expressions of p62 and LC3 II in Nrf2 WT mice, but not in Nrf2 KO mice. ^※^*p* < 0.05, ^※※^*p* < 0.01, model control versus aging control;^#^*p* < 0.05, ^##^*p* < 0.01, AP-SD versus model control; ^@@^*p* < 0.01, AP-SD 60 versus AP-SD 40. Data are means ± standard deviation (*n* = 5).

The p62 protein and LC3 have been widely regarded as markers for autophagic activity. p62 is used as a marker of autophagic degradation [30] and LC3-II serves as a marker for autophagosome formation [31]. Autophagy increases often coincides with the induction of the Nrf2 pathway in stressed conditions [32]. Based on these findings, we assessed whether Nrf2 directly upregulated the expression of p62. As shown in Figure 10(a), the upregulation of p62 expression was only found in Nrf2 WT model mice, but not in Nrf2 KO model mice, compared with the aging mice. Under the same conditions, Nrf2 levels in the nucleus significantly increased in Nrf2 WT model mice, as shown in the previous results. Our findings are consistent with those of several reports [33, 34]. In addition, AP-SD dose dependently increased p62 expression in Nrf2 WT mice, not in Nrf2KO mice (Figure 11(b)). Meanwhile, the upregulation of LC3 II expression was also only shown in Nrf2 WT mice, but not in Nrf2 KO mice, compared with the aging mice. In Nrf2 WT mice (Figure 11(a)), the expression of LC3 II increased in dose-dependent manner after treatment with AP-SD, but there was no significant change in Nrf2 KO mice (Figure 11(b)). Together, these results imply that Nrf2 is a mediator of the activation of autophagy in the model mice with dry AMD.

## 4. Discussion

Up to now, no therapy options are approved for the treatment of dry AMD. Several pathways, including oxidative stress, deposits of lipofuscin, and chronic inflammation, seem to play important roles in the pathogenesis of dry AMD and represent possible targets [35]. Oxidative stress has been suggested to be a critical component of AMD pathogenesis. Many reports demonstrated that natural products are able to decrease the occurrence of several diseases induced by oxidative injury, including AMD [36, 37]. Dietary supplements containing high dose of antioxidants and minerals (vitamins C and E, *β*-carotene, and zinc) delayed the progression of AMD intermediate to advanced stages [38]. Quercetin, lutein, zeaxanthin, and hesperetin have also been shown to have the potential to prevent AMD progress [39–41]. It has been reported that AP plays a protective role in treating diseases associated with the oxidative process, such as cardiovascular and neurological disorders [42, 43]. However, like other flavonoids, AP has poor solubility in water and fat, therefore having low bioavailability. PVP, a hydrophilic excipient, could convert drugs to an amorphous form, and immensity enhances the solubility and dissolution of drugs [44, 45]. In this study, we prepared AP-SD with PVP K30; the results showed that the formation of solid dispersion increased the solubility and dispersion of AP, so the oral bioavailability of AP-SD was better than that of AP in rats. We then used dry AMD model mouse to verify the benefits of AP-SD in improving bioavailability. The results showed that AP-SD could reduce retinopathy, while AP could not, indicating that the formation of solid dispersion reasonably enhanced the protective effects of AP on the mouse retina on account of the increased bioavailability.

Recently, the Nrf2 pathway was considered a key contributor to the response to increased oxidative stress in RPE. Nrf2 knockout mouse has been shown to induce AMD-like pathological changes, manifested by the accumulation of lipofuscin, drusen, choroidal neovascularization and accumulation of the autophagosome [46]. In this study, similar lesions were showed in the retina of the model mouse. Activation of the Nrf2 pathway could have a therapeutic potential in protecting RPE cells against oxidative stress, so it may be beneficial for dry AMD [47]. Based on this point, we have previously reported that AP protected ARPE-19 cells against oxidative injury by the activation of Nrf2 signaling *in vitro*. In the present study, the effects of AP were validated in model mouse of dry AMD *in vivo*. The upregulation of Nrf2 expression and nuclear translocation were observed in Nrf2 WT mice. AP-SD promoted Nrf2 nuclear translocation, upregulated the expression of Nrf2 and targeted protein HO-1 and GCL, restored activities of SOD and GSH-PX, and decreased the levels of ROS and MDA in Nrf2 WT mice. As expected, similar results were not observed in Nrf2 KO mice. In conclusion, our findings suggested that AP-SD protected the retina against oxidative injury via activation of the Nrf2 signaling pathway.

It was reported that AP regulate autophagy. For example, AP promoted autophagy through the mTOR/AMPK/ULK1 pathway and could play an antidepressant effect [48], inhibited the growth of cisplatin-resistant colon cancer cells by inducing autophagy and apoptosis [49], and restored impairment of autophagy and downregulation of unfolded protein response regulatory proteins in keratinocytes exposed to ultraviolet B radiation [50]. Studies have suggested that p62 protein is regulated by oxidative stress and is a transcriptional target of Nrf2 [51, 52]. This study focused on the effect of AP on autophagy pathway in the retina of model mice and the relationship between the p62 expression, autophagy, and Nrf2 pathway. The results showed that the expression of p62 and LC3 in Nrf2 KO mice were significantly lower than that in Nrf2 WT mice. In Nrf2 WT mice, AP-SD dose dependently upregulated the expression of p62 and LC3 but had no effect in Nrf2KO mice. Our finding suggested that AP-SD upregulated the autophagy in an Nrf2-dependent manner. It has been well known that the nuclear translocation of Nrf2 is increased and the expression of Nrf2 is upregulated in oxidative stress. Nrf2 could regulate the transcription of autophagy genes, such as p62, ULK1, Atg7 and GABARAPL1, Atg2B, Atg5, and Atg4D, because there is an ARE sequence in their promoter regions. The expressions of several autophagy markers (including LC3) were decreased in Nrf2-deficient mice [33, 53]. However, it has been reported that trehalose upregulate Nrf2 and autophagy in a p62-dependent manner in oxidative stress [12]. Therefore, the underlying mechanism of AP regulating the Nrf2 pathway and autophagy remains to be further studied.

In summary, AP-SD significantly enhanced the bioavailability of the original drug and reduced retinal oxidative injury in model mouse of dry AMD *in vivo*. Meanwhile, we provided evidence that AP not only activates Nrf2 pathway but also upregulates p62 and autophagy. AP-SD upregulated the expressions of antioxidant enzymes through the Nrf2 pathway and upregulated the autophagy in an Nrf2-dependent manner to suppress retinal oxidative damage. The results suggest that AP-SD is a potential compound for the treatment of dry AMD.

## Figures and Tables

**Table 1:** Equilibrium solubility of AP of both dosage forms (37°C, *n* = 3).

Solvents	Concentration (*μ*g/mL)	
	AP	AP-SD
Water	1.33 ± 0.15	352.09 ± 22.56^∗∗^
Chloroform	2.18 ± 0.21	1.43 × 10^3^ ± 187.11^∗∗^

Significance: ^∗∗^*p* < 0.01 versus AP.

**Table 2:** Pharmacokinetic parameters of AP after oral administration of pure AP and AP-SD in rats (dose 50 mg/kg, *n* = 6, mean ± SD).

Parameters	AP	AP-SD
*C*_max_ (*μ*g/mL)	0.27	1.52^∗∗^
*T*_max_ (hr)	2.0	1^∗^
AUC_0-24_ (*μ*gh/mL)	3.10	7.68^∗∗^

Significance: ^∗^*p* < 0.05, ^∗∗^*p* < 0.01 versus AP.

## Data Availability

The data used to support the findings of this study are included within the article.
